# 

**Published:** 1894-10-27

**Authors:** 


### CONTENTS.

**Sir James Crichton Browne's Cure for Quackery** - - 55

**Treatment of Perforation Peritonitis** - - - - - - 56

**Conditions of Mental Enfeeblement.**—By T. S. Clouston, M.D. - - - - - - - - - - - - - - 57

**Studies in Applied Sociology.**—"Sober by Act of Parliament" 58

**Notes and News** - - - - - - - - - - - - - 60

**Medical Progress and Hospital Clinics:**

Ingrowing Toenail and its Radical Cure - - - - - - 61

Cactus Grandiflorus: a Study in Therapeutics - - - - 62

Progress in Medicine.—Infective Diseases (*continued*) - - 63

Progress in Surgery.—Surgery of the Œsophagus and the Stomach - - - - - - - - - - - - - 65

New Appliances and Things Medical - - - - - - - 66

**Annotations.**—Barracks or Villages for Children?—Anti-Cholera Inoculation in India—The Czar's Kidney Affection - - - - 59

**The Harveian Oration** - - - - - - - - - - 66

**The Institutional Workshop:**

Canadian Hospitals.—A Visit to the Hospitals of Montreal—II. - - - - - - - - - - - - - - 67

The Story of the Insane from Year to Year - - - - 67

Practical Departments—Electric Lighting in Hospitals - - 68

Construction Notes - - - - - - - - - - - 69

**Around the Hospitals** - - - - - - - - - - 69

**Editor's Letter Box** - - - - - - - - - - 70

**Scraps and Gleanings** - - - - - - - - - - 70

**The Book World of Medicine and Science** - - - - 71

### EXTRA SUPPLEMENT.—THE NURSING MIRROR.

**News from the Nursing World.**—St. Thomas's Training School—The London Hospital—District Nursing in South London—Postponed Payments—Miss Nightingale and the Queen's Nurses—Charity at Bradford—Medical Women in Edinburgh—Skilled Nursing in Aberdeen—Fever Nurses in Ireland—Copenhagen—Midwives in Sweden—Private Nurses in Sydney—Matrimonial Contingencies—Pittsburg—Infants in Paris—Short Items - - - - - - - - - - - - xxiii

**Notes for Nurses on Antiseptic Surgery.**—By William Horrocks, M.B., F.R.C.S.—III. Ordinary Antiseptic Dressing xxv

**A New Society** - - - - - - - - - - - - xxv

**Male Nurses.**—Training in the Medical Staff Corps - - xxvii

**Matrons in Council** - - - - - - - - - - - xxvi

**Presentations—Minor Appointments** - - - - - xxvi

**Addenbrooke's Hospital, Cambridge** - - - - - xxvii

**Everybody's Opinion**, xxvii: **Dress and Uniforms** - xxviii

